# Phylogenetic analysis of simian *Plasmodium* spp. infecting *Anopheles balabacensis* Baisas in Sabah, Malaysia

**DOI:** 10.1371/journal.pntd.0005991

**Published:** 2017-10-02

**Authors:** Tock H. Chua, Benny O. Manin, Sylvia Daim, Indra Vythilingam, Chris Drakeley

**Affiliations:** 1 Department of Pathobiology and Medical Diagnostics, Faculty of Medicine and Health Sciences, Universiti Malaysia Sabah, Kota Kinabalu, Sabah, Malaysia; 2 Department of Parasitology, Faculty of Medicine, University of Malaya, Kuala Lumpur, Malaysia; 3 Faculty of Infectious and Tropical Diseases, London School of Hygiene and Tropical Medicine, London, United Kingdom; Vienna, AUSTRIA

## Abstract

**Background:**

*Anopheles balabacensis* of the Leucospyrus group has been confirmed as the primary knowlesi malaria vector in Sabah, Malaysian Borneo for some time now. Presently, knowlesi malaria is the only zoonotic simian malaria in Malaysia with a high prevalence recorded in the states of Sabah and Sarawak.

**Methodology/Principal findings:**

*Anopheles* spp. were sampled using human landing catch (HLC) method at Paradason village in Kudat district of Sabah. The collected *Anopheles* were identified morphologically and then subjected to total DNA extraction and polymerase chain reaction (PCR) to detect *Plasmodium* parasites in the mosquitoes. Identification of *Plasmodium* spp. was confirmed by sequencing the SSU rRNA gene with species specific primers. MEGA4 software was then used to analyse the SSU rRNA sequences and bulid the phylogenetic tree for inferring the relationship between simian malaria parasites in Sabah.

PCR results showed that only 1.61% (23/1,425) of the screened *An*. *balabacensis* were infected with one or two of the five simian *Plasmodium* spp. found in Sabah, viz. *Plasmodium coatneyi*, *P*. *inui*, *P*. *fieldi*, *P*. *cynomolgi* and *P*. *knowlesi*. Sequence analysis of SSU rRNA of *Plasmodium* isolates showed high percentage of identity within the same *Plasmodium* sp. group. The phylogenetic tree based on the consensus sequences of *P*. *knowlesi* showed 99.7%–100.0% nucleotide identity among the isolates from *An*. *balabacensis*, human patients and a long-tailed macaque from the same locality.

**Conclusions/Significance:**

This is the first study showing high molecular identity between the *P*. *knowlesi* isolates from *An*. *balabacensis*, human patients and a long-tailed macaque in Sabah. The other common simian *Plasmodium* spp. found in long-tailed macaques and also detected in *An*. *balabacensis* were *P*. *coatneyi*, *P*. *inui*, *P*. *fieldi* and *P*. *cynomolgi*. The high percentage identity of nucleotide sequences between the *P*. *knowlesi* isolates from the long-tailed macaque, *An*. *balabacensis* and human patients suggests a close genetic relationship between the parasites from these hosts.

## Author summary

*Anopheles balabacensis* has been incriminated as the primary vector of zoonotic simian malaria, *P*. *knowlesi* in Malaysian Borneo with a high prevalence recorded in the states of Sabah and Sarawak. In this study, *Anopheles* spp. were sampled using human landing catch (HLC) method at Paradason village in Kudat district of Sabah. Total DNA was extracted from these specimens, followed by sequencing the SSU rRNA gene of *Plasmodium* using polymerase chain reaction (PCR) for the detection and identification of *Plasmodium*. PCR results showed that only 1.61% (23/1,425) of the screened *An*. *balabacensis* had either single or double *Plasmodium* spp infections. The simian malaria parasites isolated from *An*. *balabacensis* were *P*. *coatneyi*, *P*. *inui*, *P*. *fieldi*, *P*. *cynomologi* and *P*. *knowlesi*. Sequence analysis of these *Plasmodium* isolates showed high percentage of identity within the same *Plasmodium* sp. group. Consensus sequences phylogenetic tree of *P*. *knowlesi* isolates from *An*. *balabacensis*, human patients and a long-tailed macaque from the same locality had 99.7%–100.0% nucleotide identity. This study suggests a close genetic relationship between the parasites isolated from these hosts.

## Introduction

*Anopheles* species of the Leucosphyrus group have been identified as medically important vectors in Southeast Asia region [1,2]. The Leucosphyrus group has three main subgroups; Hackeri, Leucosphyrus and Riparis subgroups [3], with the Leucosphyrus subgroup further divided into Dirus complex and Leucosphyrus complex [2,4]. In Peninsular Malaysia, three species of the Leucosphyrus group namely *An*. *hackeri*, *An*. *cracens* and *An*. *introlatus* had been incriminated as primary vectors for *P*. *knowlesi* [5–7]. However, in East Malaysia, *An*. *latens* in Sarawak and *An*. *balabacensis* in Sabah had been confirmed as primary vectors for *P*. *knowlesi* [8,9].

A study in Cambodia in 1962 has shown that *An*. *balabacensis* (identified as *An*. *dirus* later [10]) preferred biting human compared to monkeys placed at the ground level, but preferred monkeys at canopy level to monkeys on the ground [11]. A study in Sabah comparing human landing catch (HLC) and monkey baited trap (MBT) at ground level showed that more *An*. *balabacensis* were caught using HLC than MBT [12]. Recent studies showed that this species is more active during the early night with a peak biting time between 7 pm to 8 pm [9,13], and also prefers to bite outdoors than indoors [13]. Such biting behaviors coupled with an abundant source of simian malaria parasites in the reservoir long-tailed macaques (*Macaca fascicularis*) contribute to *An*. *balabacensis* becoming an effective vector for transmitting *P*. *knowlesi* malaria in Sabah.

Previous studies in Malaysia have shown that the long-tailed macaques harbor at least five species of simian *Plasmodium* [14,15], all of which have also been detected in *An*. *balabacensis* [9,16]. In Sabah, besides *P*. *knowlesi*, other simian malaria parasites recorded in *An*. *balabacensis* are *P*. *coatneyi*, *P*. *inui*, *P*. *fieldi* and *P*. *cynomolgi* [9,13]. Apart from recording these parasites in the mosquitoes, there is limited study on the phylogenetic relationship among these simian malaria parasites found in *An*. *balabacensis*, macaques and human.

In this study, we compare the partial nucleotide sequences of SSU rRNA of simian malaria parasites isolated from *An*. *balabacensis* caught in Kudat district of Sabah, from macaques as well as human patients with other published sequences of human and simian malaria parasites available in the GeneBank database. Building a phylogenetic tree of these malaria parasites will give us a clearer picture about their genetic relationship especially for *P*. *knowlesi* isolated from long-tailed macaque, *An*. *balabacensis* and human.

## Materials and methods

### Study area

**Fig 1:**
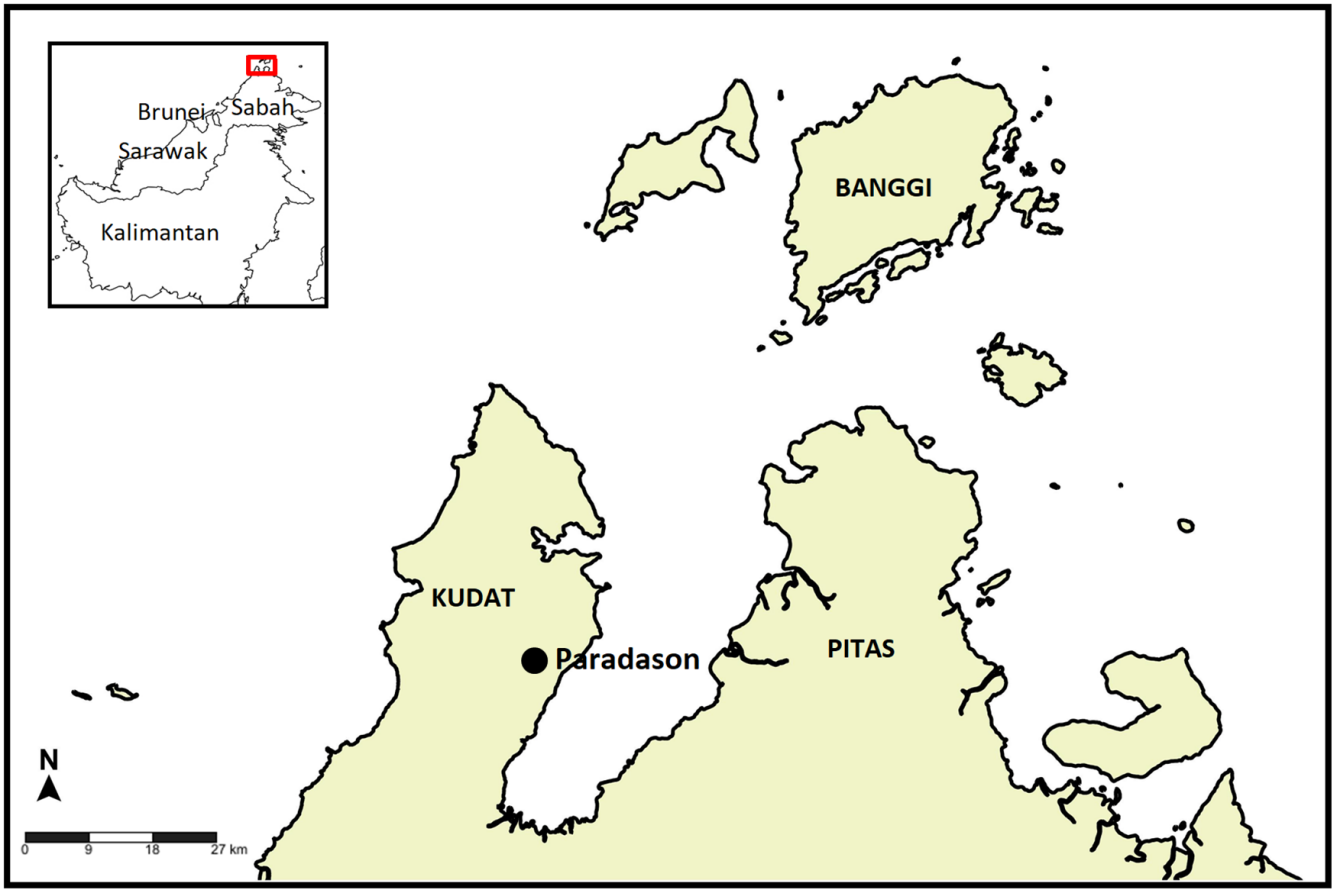
Locations of Kudat district and Paradason village at the northern tip of Sabah.

Kudat district, located at the northern tip of Borneo under the Kudat Division, is about 153 kilometers from Kota Kinabalu, the state capital of Sabah. Paradason village where the study was conducted is located in Kudat District and about 50 kilometers from Kudat town (Fig 1). Most of the villagers belong to the Rungus ethnic group who are dependent on small-scale farming (paddy), oil palm and rubber plantations as their primary source of income.

### Sampling of *Anopheles*

*Anopheles* mosquitoes were sampled monthly from October, 2013 to December, 2014 using human landing catch (HLC) method. A total 70 nights of sampling were performed starting from 1800 to 0600 hours (12 hours). Two pairs of volunteers were assigned working in shifts at a randomly selected habitat during each night of sampling. *Anopheles* was lured by the volunteers exposing their legs. The mosquitoes landing on the legs were caught by the volunteers using plastic specimen tubes (2 cm diameter X 6 cm) aided by a flashlight.

### Morphological identification of *Anopheles* species

The next morning, the *Anopheles* mosquitoes were killed by keeping them in the freezer (-20°C) for a few minutes, then gently pinned onto Nu poly strip using ultra-thin micro-headless pins. Species identification was done under a compound microscope using published keys [2,17,18]. After identification, each individual specimen was stored separately in a new microfuge tube and transported to Faculty of Medicine & Health Sciences, Universiti Malaysia Sabah for further processing.

### Total DNA extraction of *Anopheles*

Each individual *Anopheles* specimen was placed separately inside a sterilized mortar and the tissue homogenized using a sterile pestle. The total DNA was extracted from the tissues using DTAB-CTAB method [19] with some modifications (for example: incubation time was reduce to 30 minute instead of overnight and at the final step of precipitation before adding TE buffer, DNA pellet was incubated at 45°C to completely evaporate any residue of ethanol).

First, 600 μl of DTAB solution was added into the mortar and the tissue was ground using pestle until homogenized. Then, the homogenized tissue was transferred into a clean 1.5 ml microfuge tube and incubated at 68°C for 30 min. Subsequently, 600 μl of chloroform was added into the microfuge tube which was inverted ten times to mix the contents and centrifuged at 13,000 rpm for 5 min. Then, 400 μl of the upper aqueous layer was carefully transferred into a new clean 1.5 ml microfuge tube and mixed with 900 μl sterile dH_2_O and 100 μl CTAB solution by gently inverting the microfuge tube for several times and allowed it to sit at room temperature for 5 min. The mixture was then spun at 13,000 rpm for 10 min. The supernatant was discarded and the DNA pellet was re-suspended in 300 μl of 1.2 M NaCl solution. Total DNA was precipitated by adding 750 μl of absolute ethanol and centrifuged at 13,000 rpm for 5 min. The supernatant was discarded, the DNA pellet washed with 500 μl of 70% ethanol and centrifuged at 13,000 rpm for 2 min. The DNA pellet was incubated at 45°C for 10 min and re-suspended in 30 μl Tris-EDTA (pH8.0) buffer and stored at -30°C.

### Amplification of *Plasmodium* DNA

Presence of malaria parasites in the mosquitoes was detected using nested PCR by targeting the small subunit ribosomal RNA (SSU rRNA) gene of *Plasmodium*. A PCR primer pair, rPLU1 and rPLU5, was used in first PCR reaction, while another pair (rPLU3 and rPLU4) was used in the second PCR reaction [20]. For internal control, another set of nested PCR was performed separately to amplify the cytochrome c oxidase subunit II (COII) gene of *Anopheles* [12]. When a mosquito was confirmed positive for malaria parasites, the *Plasmodium* species was determined using species specific primers. Both PCR reactions were performed with 25.0 μl final volume.

The reaction components were prepared by mixing 5.0 μl of 5X PCR buffer (Promega), 0.5 μl of (10 mM) dNTPs (Promega), 3.0 μl of (25 mM) MgCl_2_, 1.0 μl of (10 μM) forward and reverse primers, 0.3 μl of (5.0 U/μl) Taq DNA polymerase (Promega), 2.0 μl of DNA template and sterile dH_2_O to make up to 25.0 μl final volume. After completion of the first PCR, 2.0 μl of the PCR product was used as DNA template in the second PCR. The reaction was carried out using a thermal cycler (T100 Thermal Cycler, BioRad) with an initial denaturation at 95°C for 5 min followed by 35 cycles of denaturation at 94°C for 1 min, annealing for 1 min and extension at 72°C for 1 min and one final extension step at 72°C for 5 min. The annealing temperature was set at optimal temperature for each set of primers (see S1 Table). The PCR products were analyzed on 1.5% agarose gel electrophoresis stained with RedSafe nucleic acid staining solution (iNtRON Biotechnology), and visualized with an UV transilluminator.

### Cloning and sequencing of SSU rRNA gene of simian *Plasmodium*

The SSU rRNA gene of the five simian malaria parasite species extracted from *An*. *balabacensis* caught in Paradason were cloned and sequenced. In addition, we included in the study blood samples from two *P*. *knowlesi* patients and two long tail macaques, one infected with *P*. *knowlesi* while the other with *P*. *inui*. To make the data set larger, we included simian malaria parasites obtained from mosquitoes caught in three other villages (Tomohon, Mambatu Laut and Narandang) in Kudat district from another study.

A new universal forward primer (UMSF) combined with species-specific primers were used to amplify the SSU rRNA gene of *Plasmodium*. Details of the primers are provided in S2 Table. Preparation of the reaction mixture and the PCR conditions programmed are as described above. After the PCR was completed, the PCR products were purified to remove impurity and excess reaction mixture using MEGA quick-spin PCR & Agarose Gel DNA Extraction System (iNtRON Biotechnology, Korea) according to manufacturer’s procedure.

Cloning the SSU rRNA gene was done using pGEM-TEasy vectors (Promega, USA) and the plasmids were extracted from the transformed *E*. *coli* (JM109) using DNA-spin Plasmid DNA Purification Kit (iNtRON Biotechnology, Korea), all according to the manufacturer’s protocol. The extracted plasmid vectors were restricted using EcoRI restriction enzyme (Promega, USA) and sent to AITBIOTECH, Singapore for sequencing. Sequencing was carried in both directions using forward and reverse M13 primers.

### BLAST search of SSU rRNA sequence

The nucleotide sequences of SSU rRNA of 21 *Plasmodium* isolates in this study were aligned and compared with other SSU rRNA sequences available at the GeneBank database to determine the percentage identity using Basic Local Alignment Search Tool (BLAST) available online at https://blast.ncbi.nlm.nih.gov/Blast.

### Sequence analysis and phylogenetic tree of SSU rRNA

The SSU rRNA sequences were standardized to a fixed region for analysis based on the UMSF and UNR primers binding sites. Further analysis was performed using MEGA software, version 4.1 [21]. The nucleotide sequences were multi-aligned using ClustalW method [22] incorporated in the software and the number of variable nucleotides within each of the five *Plasmodium* spp. determined.

Phylogenetic tree was constructed using neighbor-joining method [23] and the evolutionary distances computed using maximum composite likelihood model with a bootstrap test of 1000 replicates [24] and pairwise deletion option. This method was adopted as it takes into account the different rates of evolution or substitution between nucleotides. The selected region for constructing the phylogenetic tree was nucleotides numbered nt81 to nt1041, based on the published *P*. *knowlesi* sequence (AY327551) isolated in Kapit Sarawak where there was a large focus of infected people [25]. This region includes the binding sites for universal forward (UMSF, used in this study) and reverse primers (UNR, [26]) of SSU rRNA. In constructing the phylogenetic tree, *Theileria* spp. (AF162432) was used as the outgroup. Details of the other 66 nucleotide sequences that were used in constructing the phylogenetic tree are given in S3 Table. Both *Plasmodium simium* (AY579415) and *P*. *brasilianum* (AF130735, KT266778) were not included in the sequence analysis as the selected sequence used in this study was not available in GeneBank database.

A second phylogenetic tree was constructed using the consensus sequences of five *Plasmodium* species found in Sabah to show the relationship between *Plasmodium* isolates found in the macaque, *An*. *balabacensis* and human.

### Ethical clearance

This project was approved by the National Medical Ethics Committee (NMRR), Ministry of Health Malaysia (Ref. NMRR-12-786-13048). All volunteers who carried out mosquito collections signed informed consent forms and were provided with antimalarial prophylaxis during the study period. Blood spots on Whatman filter paper were collected from adult patients by Kudat hospital personnel, after they had signed informed consent forms. This human blood sample collection was also approved by the NMRR (Ref. NMRR–11–4539471). Blood spots on filter paper were collected by wild life department personel from ten wild macaques captured for relocation purposes and kept in cages following the guidelines in the Animals (Scientific Procedures) Act 1986 Code of Practice for the Housing and Care of Animals Used in Scientific Procedures (UK), with the approval from the London School of Hygiene and Tropical Medicine Animal Welfare and Ethical Review Body (AWER, Ref.2012/8N). Fecal samples were not used then as the protocol for storing the samples had not yet been established by primatology group of the research team.

## Results

### Abundance of *Anopheles* species

A total of 1,599 *Anopheles* individuals belonging to ten species were caught during 14 months of sampling (Table 1). *Anopheles balabacensis* was the dominant species in Paradason village comprising 89.87% of the total catch, followed by *An*. *barbumbrosus* (5.75%), *An*. *maculatus* (1.38%) and *An*. *donaldi* (1.19%).

**Table 1:** *Anopheles* species caught at Paradason village from October 2013 to December 2014 during a total of 70 human sampling nights.

Series/Group	Species	Total number	Total %
Leucosphyrus	*An*. *balabacensis*	1,437	89.87
Barbirostris	*An*. *barbumbrosus*	92	5.75
	*An*. *donaldi*	19	1.19
Maculatus	*An*. *maculatus*	22	1.38
Hycanus	*An*. *nigerrimus*	5	0.31
	*An*. *peditaeniatus*	4	0.25
Umbrosus	*An*. *separates*	1	0.06
	*An*. *umbrosus*	3	0.19
Pyretophorus	*An*. *sundaicus*	2	0.13
Tessellatus	*An*. *tessellates*	14	0.88
	Total	1,599	100.00

### Infection of *Anopheles* specimens with malaria parasites

A total of 1,586 *Anopheles* mosquitoes (of which 1,425 were *An*. *balabacensis*) were tested for presence of malaria parasites using the PCR method. Only 23 *An*. *balabacensis* (1.61%) were found to have malaria parasites in them, being infected with one (78.3%) or two simian *Plasmodium* spp. (Table 2). The single infection was mostly by *P*. *inui* (n = 11).

**Table 2:** Diversity of simian *Plasmodium* species in infected *An*. *balabacensis* detected using PCR method.

*Plasmodium* spp.	Number of *An*. *balabacensis*	%	Time mosquitoes caught
*P*. *inui*	11	47.8	6–7 pm (n = 2); 7–8 pm (n = 1); 8–9 pm (n = 2); 9–10 pm (n = 3); 1–2 am (n = 2); 3–4 am (n = 1)
*P*. *cynomolgi*	4	17.4	9–10 pm (n = 1); 12–1 am (n = 2); 2–3 am (n = 1)
*P*. *coatneyi*	3	13.0	6–7 pm (n = 1); 11–12 am (n = 1); 3–4 am (n = 1)
*P*. *inui* + *P*. *cynomolgi*	2	8.7	6–7 pm (n = 1); 3–4 am (n = 1)
*P*. *knowlesi* + *P*. *inui*	1	4.4	11–12 am (n = 1)
*P*. *knowlesi* + *P*. *cynomolgi*	1	4.4	7–8 pm (n = 1)
*P*. *fieldi* + *P*. *cynomolgi*	1	4.4	10–11 pm (n = 1)

### BLAST search of SSU rRNA sequences of *Plasmodium* spp.

BLAST analysis of 21 SSU rRNA sequences of *Plasmodium* spp. isolated from *An*. *balabacensis*, human and long tail macaques (3 samples of *P*. *coatneyi*, 1027–1029 bp; 4 samples of *P*. *cynomolgi*, 1015 bp; 3 of *P*. *fieldi*, 1039 bp; 6 of *P*. *inui*, 1039 bp and 5 of *P*. *knowlesi*, 1050 bp) showed high percentage of identity with the simian *Plasmodium* nucleotide sequences published in the GeneBank database.

The *Plasmodium* species in Sabah show a high percentage identity within the same species groups (98.4%–99.6%) but less between different species groups. The highest percentage identity (99.6%) was observed between the *P*. *cynomolgi* samples isolated from Tomohon, Membatu Laut and Paradason villages, while the least was for *P*. *coatneyi* isolates (98.4%) obtained from Narandang and Paradason villages.

The SSU rRNA sequences of *Plasmodium* spp. from Sabah also show high percentage identity with the same species from other Asian regions. *Plasmodium coatneyi* sequences showed 99% identity with *P*. *coatneyi* isolated from *M*. *fascicularis* in Kapit, Sarawak (FJ619094), as well as with CDC (AB265790) and Hackeri (CP016248) strains. *Plasmodium cynomolgi* sequences showed 99%–100% identity with *P*. *cynomolgi* isolated from *M*. *fascicularis* in Kapit, Sarawak (FJ619084), and from other macaque species viz. *M*. *radiata* (AB287290) of southern India and *M*. *nemestrina* (AB287289) from unspecified South-east Asian nation. Similarly, *P*. *fieldi* has high percentage identity with *P*. *fieldi* isolated from *M*. *fascicularis* in Kapit, Sarawak (KC662444). Of interest is *P*. *inui*, which not only has high identity (99%–100%) with those isolated in Kapit (FJ619074) but also with *P*. *inui* isolated from *M*. *fascicularis* from South China (HM032051), Southern Thailand (EU400388) and strain Taiwan II isolate from *M*. *cyclopis* (FN430725).

The *P*. *knowlesi* samples of Sabah showed 99% identity with *P*. *knowlesi* isolated from both human (AY327551) and *M*. *fascicularis* (FJ619089) in Kapit, Sarawak, as well as with that from a Swedish traveler who was infected during his visit to Sarawak (EU807923) [27].

### Sequence analysis and phylogenetic trees

The number of nucleotides in the analyzed region for the various *Plasmodium* spp. are: *P*. *knowlesi* 961 bp, *P*. *inui* 946, *P*. *coatneyi* 942, *P*. *cynomolgi* 935 and *P*. *fieldi* 934 respectively. Sequence alignment indicated that *P*. *coatneyi* has the highest number of variable nucleotides among the isolates (n = 3 isolates; 15 variable nucleotides) followed by *P*. *knowlesi* (n = 5; 9), *P*. *inui* (n = 6; 7), *P*. *fieldi* (n = 3; 5) and *P*. *cynomolgi* (n = 4; 4).

**Fig 2:**
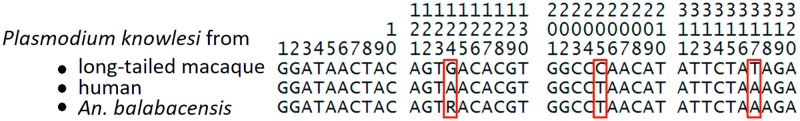
Consensus sequences of SSU rRNA of *P*. *knowlesi* isolated from long-tailed macaque, human and *An*. *balabacensis* in Sabah. The three variable nucleotides in the consensus sequences between the *P*. *knowlesi* isolates are shown within the red rectangles.

Further analysis of the *P*. *knowlesi* group using consensus sequences showed that there were three variable nucleotides between *P*. *knowlesi* isolated from the long-tailed macaque and human, two between long-tailed macaque and *An*. *balabacensis* isolates but none between *An*. *balabacensis* and human isolates (Fig 2).

**Fig 3:**
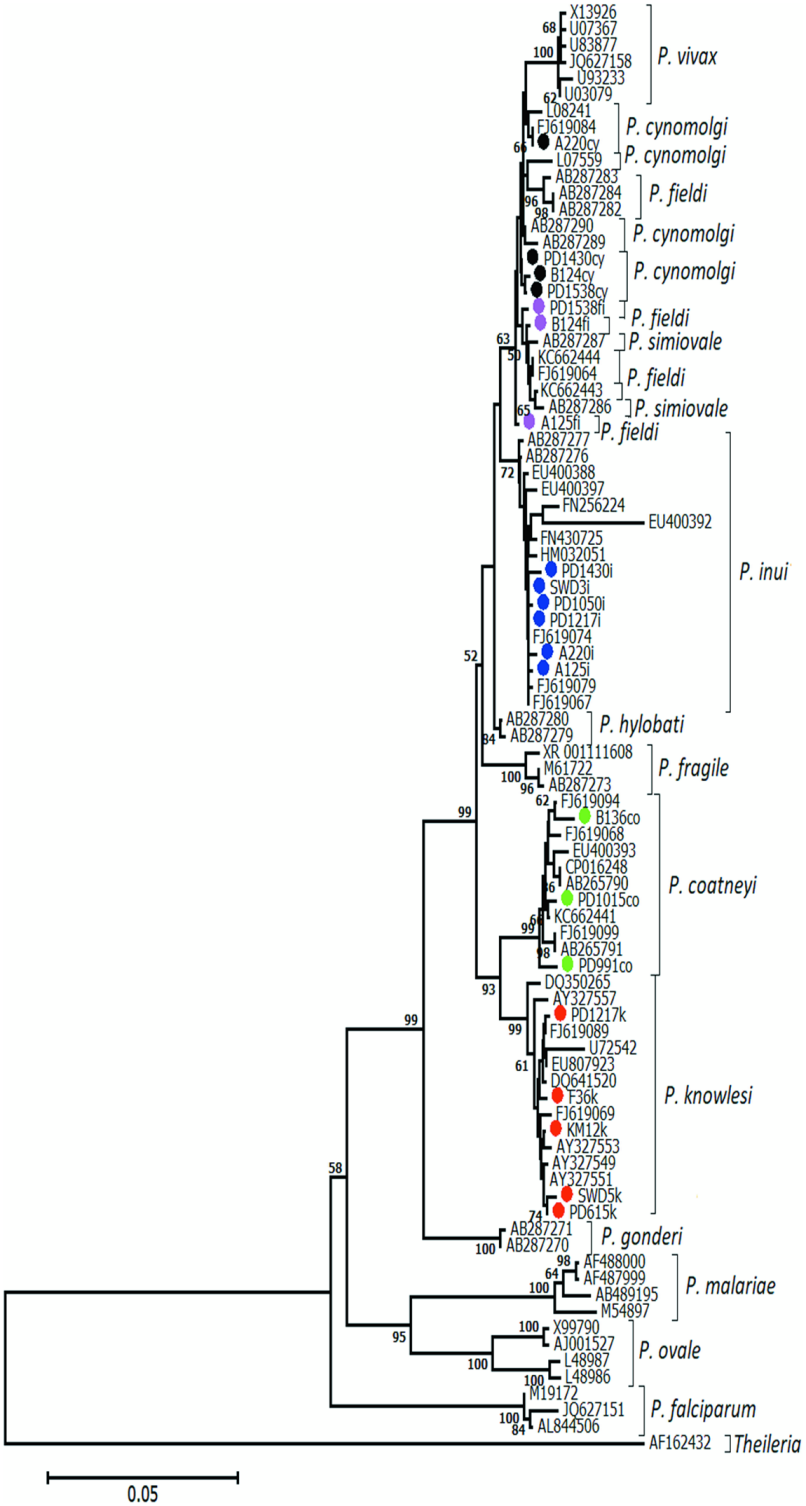
Neighbor-joining phylogenetic tree comparing the SSU rRNA gene sequences in current study (marked with circle) and with known *Plasmodium* SSU rRNA sequences from the GeneBank data base. The bar below the tree represents distance scale. The evolutionary distances were computed using the maximum composite likelihood method and all positions containing alignment gaps and missing data were eliminated only in the pairwise sequence comparisons. The tree was replicated with 1000 bootstraps and only values>50% are showed in the tree. The tree was out grouped with *Theileria* spp. (AF162432).

In the phylogenetic tree generated for 13 *Plasmodium* species infecting monkeys and humans (Fig 3), all the 21 *Plasmodium* isolates obtained in the study were placed in the correct species group. *P*. *knowlesi* group was positioned below *P*. *coatneyi* group whereas *P*. *inui*, *P*. *fieldi* and *P*. *cynomolgi* were placed at the upper branches.

**Fig 4:**
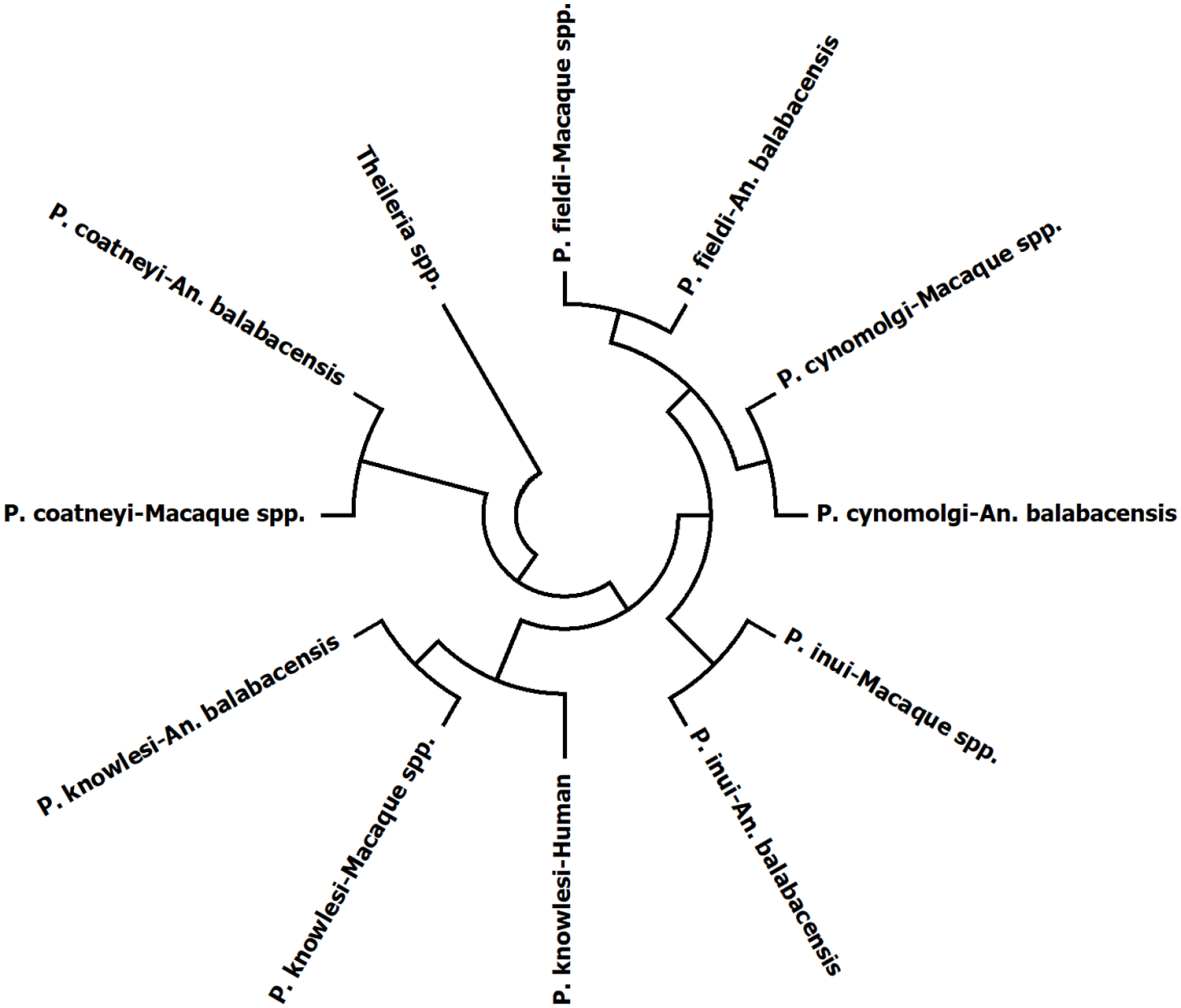
Phylogenetic tree based on consensus sequences of SSU rRNA showing the relationship between the *Plasmodium* species in Sabah that were found in monkey, *An*. *balabacensis* and human, constructed using neighbor joining method. The evolutionary distances were computed using the maximum composite likelihood method and all positions containing alignment gaps were eliminated only in the pairwise sequence comparisons. The tree was out grouped with *Theileria* spp.

In the phylogenetic tree depicting relationship between the five *Plasmodium* species found in Sabah using consensus sequences, a similar tree topology was also observed (Fig 4). All *Plasmodium* group except for *P*. *knowlesi* group has two branches, each representing the host from which *Plasmodium* was isolated. However, *P*. *knowlesi* group has three branches with the isolates from both *An*. *balabacensis* and macaque closer to each other than to the isolates from humans.

## Discussion

In this study, we analyzed 21 nucleotide sequences of partial SSU rRNA of five *Plasmodium* spp. isolated from *An*. *balabacensis* collected in Kudat district of Sabah, infected humans and a long-tailed macaque together with other nucleotide sequences downloaded from the GeneBank database. The results suggest that in Sabah, there is a close genetic relationship between the *P*. *knowlesi* specimens in the long-tailed macaques, *An*. *balabacensis* and human.

*Plasmodium inui* appears to be a common simian malaria parasite found in 61% (14/23) of the infected *An*. *balabacensis* specimens. This was also the case in other investigations [9,28]. Hitherto, this simian malaria has not become zoonotic to humans yet although it has been proven experimentally to be infective to monkey through the bites of *An*. *dirus* [29]. The infection rate of *P*. *knowlesi* in *An*. *balabacensis* is low (0.14%, 2/1,425) with only two mosquitoes being infected along with other *Plasmodium* species. Nevertheless *P*. *knowlesi* is the dominant *Plasmodium* species recorded among the human cases in Sabah [30]. These cases were recorded mainly in the rural areas near to forests and also among the workers in the agricultural sector viz. in oil palm estates and vegetable farms [13,31].

Sequence data of the SSU rRNA of *Plasmodium* confirm that the five species of simian *Plasmodium* commonly harbored by the wild macaques in Malaysia are also found in *An*. *balabacensis*. BLAST results of Sabah’s *Plasmodium* sequences showed high identity with other simian *Plasmodium* sequences published in the GeneBank database, especially with the simian malaria parasites in long-tailed macaques in Kapit, Sarawak (FJ619069 and FJ619089). This could suggest that a similar or closely related cluster of simian *Plasmodium* is circulating among the monkey populations and *Anopheles* mosquitoes in both Sabah and Sarawak. This is highly plausible as these two states share a common boundary, and there is a continual movement of humans between these two states.

The total number of nucleotides in the analyzed region was different for the five simian *Plasmodium* spp. in Sabah, with *P*. *knowlesi* having a higher number. The differences in total number of nucleotides in the SSU rRNA gene confer a unique signature to each *Plasmodium* species. Furthermore the presence of conserved and variable sequences in the gene makes it suitable for species identification and phylogenetic study [32,33].

The percentage of identity between consensus sequences of SSU rRNA of *P*. *knowlesi* isolates from the monkey, mosquito and man was high (Fig 2). For example, 100% identity was observed between *P*. *knowlesi* isolates from *An*. *balabacensis* and human, 99.8% between *An*. *balabacensis* and the long-tailed macaque, and 99.7% between long-tailed macaque and human. This indicates a great genetic similarity in *P*. *knowlesi* found in the long-tailed macaque, *An*. *balabacensis* and human populations. However, it is not certain if this would indicate the same cluster of *P*. *knowlesi* is circulating between these hosts, since we did not dissect the mosquitoes’ salivary glands to detect for sporozoites, or carry out RT-PCR targeting the specific mRNA transcripts of the sporozoite stage. Thus further study is needed to determine this, using more *P*. *knowlesi* positive *Anopheles balabacensis* and analyzing other polymorphic markers or microsatellite loci of the parasite. Different *P*. *knowlesi* haplotypes have been observed in the macaque and human populations in Kapit Sarawak [14] as well as in the human population in Thailand [34].

Overall, the 13 *Plasmodium* species in the phylogenetic tree can be grouped into two main clusters, one containing the *P*. *vivax*/simian malaria parasites while the other human malaria parasites (Fig 3). Although *P*. *simium* (AY579415) and *P*. *brasilianum* (AF130735, KT266778) were not included in our analysis as their nucleotide sequences in the GeneBank database do not contain the same analyzed region, *P*. *simium* is closely related to *P*. *vivax* [32]and can be placed in the first cluster, while *P*. *brasilianum* is closely related to *P*. *malariae* and can be placed in the second cluster. It may be noted that *P*. *cynomolgi*, *P*. *fieldi* and *P*. *simiovale* were not clearly resolved as some of the isolates were grouped in different branches. This could be due to the high percentage of nucleotide identity (99.6%) among these three species.

The consensus tree (Fig 4) of *Plasmodium* species found in Sabah showed a very close relationship between the *Plasmodium* isolates from monkey as the reservoir, *An*. *balabacensis* as the vector, and human as the case. This is supported by *P*. *knowlesi* isolates from these three organisms having high nucleotide identity (99.7–100%).

Currently in Sabah, *An*. *balabacensis* is the only species found to carry *P*. *knowlesi*. The phylogenetic analysis here indicates that the vector picks up the malaria parasites from monkeys and transmits them to humans when it feeds on them. However, there is a lot more about the transmission dynamics of *P*. *knowlesi* that is still unknown and needs to be unpacked. A clearer picture on the interrelationship of simian malaria parasites found in *An*. *balabacensis* will help us to understand more about *Plasmodium* itself. Future research may focus more on the host-vector relationship that requires longer nucleotide sequence analysis so that new informed alternatives for malaria elimination strategy targeting on *P*. *knowlesi* as well as other simian malaria parasites may be formulated.

## Supporting information

S1 Table Details of PCR primers used in PCR reactions for detection of *Plasmodium* parasites in *Anopheles* specimens. (DOCX)

S2 Table Forward and reverse PCR primers used to amplify partial region SSU rRNA of five *Plasmodium* species extracted from *An*. *balabacensis*. The amplified region was cloned and sequenced for further analysis. (DOCX)

S3 Table Information on nucleotide sequences of SSU rRNA gene downloaded from GeneBank database used in building phylogenetic tree. (DOCX)
