# Supplementary material for: Phylogenetic analysis of simian Plasmodium spp. infecting Anopheles balabacensis Baisas in Sabah, Malaysia
Source: PLoS Negl Trop Dis. 2017 Oct 2;11(10):e0005991. doi: 10.1371/journal.pntd.0005991 (PMC5638607; doi:10.1371/journal.pntd.0005991)
Supplement: S1 Table — (DOCX) [file pntd.0005991.s001.docx]

Supplementary Table 1: Details of PCR primers used in PCR reactions for detection of *Plasmodium* parasites in *Anopheles* specimens.

| Target | Genus / species | Primer set for PCR | Set primer | Primer name | Reference | Sequence (5’ - 3’) | Annealing temperature (^o^C) | Size of PCR product (bp) |
| --- | --- | --- | --- | --- | --- | --- | --- | --- |
| SSU-rRNA | *Plasmodium* genus | Sets 1 + 2 | Set 1 | rPLU1 | Singh *et al*., 1999 | TCAAAGATTAAGCCATGCAAGTGA | 55 | 1640 |
|  |  |  |  | rPLU5 | Singh *et al*., 1999 | CCTGTTGTTGCCTTAAACTCC |  |  |
|  |  |  | Set 2 | rPLU3 | Singh *et al*., 1999 | TTTTTATAAGGATAACTACGGAAAAGCTGT | 62 | 240 |
|  |  |  |  | rPLU4 | Singh *et al*., 1999 | TACCCGTCATAGCCATGTTAGGCCAATACC |  |  |
| COII | *Anopheles* genus | Sets 3 + 4 | Set 3 | COIIF | Yang *et al*., 2011 | TCTAATATGGCAGATTAGTGCA | 55 | 791 |
|  |  |  |  | X2R | Hawkes *et al*., 2017 | TGATTTAAGAGATCATTACTTGC |  |  |
|  |  |  | Set 4 | X2F | Hawkes *et al*., 2017 | GGCAGATTAGTGCAATGAATT | 55 | 766 |
|  |  |  |  | COIIR | Yang *et al*., 2011 | ACTTGCTTTCAGTCATCTAATG |  |  |
| SSU-rRNA | *P. coatneyi* | Sets 1 + 5 | Set 5 | PctF1 | Lee *et al*., 2011 | CGCTTTTAGCTTAAATCCACATAACAGAC | 62 | 504 |
|  |  |  |  | PctR1 | Lee *et al*., 2011 | GAGTCCTAACCCCGAAGGGAAAGG |  |  |
|  | *P. inui* | Sets 1 + 6 | Set 6 | PinF2 | Lee *et al*., 2011 | CGTATCGACTTTGTGGCATTTTTCTAC | 60 | 479 |
|  |  |  |  | INAR3 | Lee *et al*., 2011 | GCAATCTAAGAGTTTTAACTCCTC |  |  |
|  | *P. fieldi* | Sets 1 + 7 | Set 7 | PfldF1 | Lee *et al*., 2011 | GGTCTTTTTTTTGCTTCGGTAATTA | 66 | 421 |
|  |  |  |  | PfldR2 | Lee *et al*., 2011 | AGGCACTGAAGGAAGCAATCTAAGAGTTTC |  |  |
|  | *P. cynomolgi* | Sets 1 + 8 | Set 8 | CY2F | Lee *et al*., 2011 | GATTTGCTAAATTGCGGTCG | 60 | 137 |
|  |  |  |  | CY4R | Lee *et al*., 2011 | CGGTATGATAAGCCAGGGAAGT |  |  |
|  | *P. knowlesi* | Sets 1 + 9 | Set 9 | PkF1140 | Imwong *et al*., 2009 | GATTCATCTATTAAAAATTTGCTTC | 50 | 424 |
|  |  |  |  | PkR1550 | Imwong *et al*., 2009 | GAGTTCTAATCTCCGGAGAGAAAAGA |  |  |
|  | *P. falciparum* | Sets 1 + 10 | Set 10 | NewPLFshort | Ta *et al*., 2014 | CTATCAGCTTTTGATGTTAG | 53 | 370 |
|  |  |  |  | FARshort | Ta *et al*., 2014 | GTTCCCCTAGAATAGTTACA |  |  |
|  | *P. vivax* | Sets 1 + 11 | Set 11 | NewPLFshort | Ta *et al*., 2014 | CTATCAGCTTTTGATGTTAG | 53 | 476 |
|  |  |  |  | VIRshort |  | AAGGACTTCCAAGCC |  |  |
|  | *P. malariae* | Sets 1 + 12 | Set 12 | NewPLFshort | Ta *et al*., 2014 | CTATCAGCTTTTGATGTTAG | 53 | 241 |
|  |  |  |  | MARshort | Ta *et al*., 2014 | TCCAATTGCCTTCTG |  |  |
|  | *P. ovale* | Sets 1 + 13 | Set 13 | NewPLFshort | Ta *et al*., 2014 | CTATCAGCTTTTGATGTTAG | 53 | 407 |
|  |  |  |  | OVRshort | Ta *et al*., 2014 | AGGAATGCAAAGARCAG |  |  |

1. Singh, B., et al., *A genus- and species-specific nested polymerase chain reaction malaria detection assay for epidemiologic studies.* American Journal of Tropical Medicine and Hygiene, 1999. **60**(4): p. 687-692.
2. Yang, M.N., Y.J. Ma, and J. Wu, *Mitochondrial genetic differentiation across populations of the malaria vector Anopheles lesteri from China (Diptera: Culicidae).* Malaria Journal, 2011. **10**.
3. Hawkes, F., et al., *Evaluation of electric nets as means to sample mosquito vectors host-seeking on humans and primates*. Parasites & Vectors 2017, **10**.
4. Lee, K.S., et al., *Plasmodium knowlesi: reservoir hosts and tracking the emergence in humans and macaques.* Plos Pathogens, 2011. **7**(4).
5. Imwong, M., et al., *Spurious amplification of a Plasmodium vivax small-subunit RNA gene by use of primers currently used to detect P. knowlesi.* Journal of Clinical Microbiology, 2009. **47**(12): p. 4173-4175.
6. Ta, T.H., et al., *First case of a naturally acquired human infection with Plasmodium cynomolgi.* Malaria Journal, 2014. **13**.
