# Supplementary material for: Phylogenetic analysis of simian Plasmodium spp. infecting Anopheles balabacensis Baisas in Sabah, Malaysia
Source: PLoS Negl Trop Dis. 2017 Oct 2;11(10):e0005991. doi: 10.1371/journal.pntd.0005991 (PMC5638607; doi:10.1371/journal.pntd.0005991)
Supplement: S2 Table — (DOCX) [file pntd.0005991.s002.docx]

Supplementary Table 2: Combination of forward and reverse PCR primers used to amplify partial region SSU rRNA of five *Plasmodium* species. The amplified region was clone and sequence.

| *Plasmodium* spp. | Primer name | Reference | Primer sequence (5’ - 3’) | Annealing temperature (^o^C) | Size of PCR product (bp) |
| --- | --- | --- | --- | --- | --- |
| *P. coatneyi* | UMSF | This study | GGATAACTACGGAAAAGCTGT | 55 | 1030 |
|  | PctR1 | Lee *et al*., 2011 | GAGTCCTAACCCCGAAGGGAAAGG |  |  |
| *P. inui* | UMSF | This study | GGATAACTACGGAAAAGCTGT | 55 | 1048 |
|  | INAR3 | Lee *et al*., 2011 | GCAATCTAAGAGTTTTAACTCCTC |  |  |
| *P. fieldi* | UMSF | This study | GGATAACTACGGAAAAGCTGT | 55 | 1039 |
|  | PfldR2 | Lee *et al*., 2011 | AGGCACTGAAGGAAGCAATCTAAGAGTTTC |  |  |
| *P. cynomolgi* | UMSF | This study | GGATAACTACGGAAAAGCTGT | 55 | 1015 |
|  | CYN1R | This study | GATTAACTCCGAAGAGAAAATC |  |  |
| *P. knowlesi* | UMSF | This study | GGATAACTACGGAAAAGCTGT | 55 | 1050 |
|  | PkR1550 | Imwong *et al*., 2009 | GAGTTCTAATCTCCGGAGAGAAAAGA |  |  |

1. Lee, K.S., et al., *Plasmodium knowlesi: reservoir hosts and tracking the emergence in humans and macaques.* Plos Pathogens, 2011. **7**(4).
2. Imwong, M., et al., *Spurious amplification of a Plasmodium vivax small-subunit RNA gene by use of primers currently used to detect P. knowlesi.* Journal of Clinical Microbiology, 2009. **47**(12): p. 4173-4175.
