# Supplementary material for: Phylogenetic analysis of simian Plasmodium spp. infecting Anopheles balabacensis Baisas in Sabah, Malaysia
Source: PLoS Negl Trop Dis. 2017 Oct 2;11(10):e0005991. doi: 10.1371/journal.pntd.0005991 (PMC5638607; doi:10.1371/journal.pntd.0005991)
Supplement: S3 Table — (DOCX) [file pntd.0005991.s003.docx]

Supplementary Table 3: Information on nucleotide sequences of SSU rRNA gene downloaded from GeneBank database used in building phylogenetic tree.

| Species | Host/vector/isolate source | Region/country | Accession number |
| --- | --- | --- | --- |
| *P. coatneyi* | *Macaca fascicularis* | Philippines | AB265790 |
| *P. coatneyi* | *M. fascicularis* | Philippines | AB265791 |
| *P. coatneyi* | *M. mulatta* (Lab) | USA | CP016248 |
| *P. coatneyi* | *M. fascicularis* | Thailand | EU400393 |
| *P. coatneyi* | *M. fascicularis* | Malaysia | FJ619068 |
| *P. coatneyi* | *M. fascicularis* | Malaysia | FJ619094 |
| *P. coatneyi* | *M. fascicularis* | Malaysia | FJ619099 |
| *P. coatneyi* | *M. fascicularis* | Malaysia | KC662441 |
| *P. cynomolgi* | *M. nemestrina* | Southeast Asia | AB287289 |
| *P. cynomolgi* | *M. radiata* | Southeast Asia | AB287290 |
| *P. cynomolgi* | *M. fascicularis* | Malaysia | FJ619084 |
| *P. cynomolgi* | *M. mulatta* (Lab) | -- | L07559 |
| *P. cynomolgi* | *M. mulatta* (Lab) | -- | L08241 |
| *P. falciparum* | -- | UK | AL844506 |
| *P. falciparum* | -- | India | JQ627151 |
| *P. falciparum* | *Homo sapiens* | -- | M19172 |
| *P. fieldi* | *M. fascicularis* | Malaysia | AB287282 |
| *P. fieldi* | *M. fascicularis* | Malaysia | AB287283 |
| *P. fieldi* | *M. fascicularis* | Malaysia | AB287284 |
| *P. fieldi* | *M. fascicularis* | Malaysia | FJ619064 |
| *P. fieldi* | *M. fascicularis* | Malaysia | KC662443 |
| *P. fieldi* | *M. fascicularis* | Malaysia | KC662444 |
| *P. fragile* | *M. sinisca* | Sri Lanka | AB287273 |
| *P. fragile* | -- | -- | M61722 |
| *P. fragile* | -- | -- | XR_001111608 |
| *P. gonderi* | *Cercopithecus* spp. | Central Africa | AB287270 |
| *P. gonderi* | *Cercopithecus* spp. | Central Africa | AB287271 |
| *P. hylobati* | *Hylobati moloch* | Malaysia | AB287279 |
| *P. hylobati* | *H. moloch* | Malaysia | AB287280 |
| *P. inui* | *M. cyclopis* | South and East Asia | AB287276 |
| *P. inui* | *M. fascicularis* | South and East Asia | AB287277 |
| *P. inui* | *M. fascicularis* | Thailand | EU400388 |
| *P. inui* | *M. fascicularis* | Thailand | EU400392 |
| *P. inui* | *M. fascicularis* | Thailand | EU400397 |
| *P. inui* | *M. fascicularis* | Malaysia | FJ619067 |
| *P. inui* | *M. fascicularis* | Malaysia | FJ619074 |
| *P. inui* | *M. fascicularis* | Malaysia | FJ619079 |
| *P. inui* | *M. cyclopis* | Taiwan | FN256224 |
| *P. inui* | *M. mulatta* (Lab) | Taiwan | FN430725 |
| *P. inui* | *M. fascicularis* | China | HM032051 |
| *P. knowlesi* | *H. sapiens* | Malaysia | AY327549 |
| *P. knowlesi* | *H. sapiens* | Malaysia | AY327551 |
| *P. knowlesi* | *H. sapiens* | Malaysia | AY327553 |
| *P. knowlesi* | *M. mulatta* (Lab), Nuri strain | Malaysia | AY327557 |
| *P. knowlesi* | *M. fascicularis* | Malaysia | DQ350265 |
| *P. knowlesi* | *M. fascicularis* | Malaysia | DQ641520 |
| *P. knowlesi* | *H. sapiens* | Sweden | EU807923 |
| *P. knowlesi* | *M. fascicularis* | Malaysia | FJ619069 |
| *P. knowlesi* | *M. fascicularis* | Malaysia | FJ619089 |
| *P. knowlesi* | *M. mulatta* (Lab) | -- | U72542 |
| *P. malariae* | *Pan troglodytes* | Japan | AB489195 |
| *P. malariae* | *H. sapiens* | Myanmar | AF487999 |
| *P. malariae* | *H. sapiens* | Myanmar | AF488000 |
| *P. malariae* | *H. sapiens* | Uganda | M54897 |
| *P. ovale* | *H. sapiens* | Cameroon | AJ001527 |
| *P. ovale* | *H. sapiens* | -- | L48986 |
| *P. ovale* | *H. sapiens* | -- | L48987 |
| *P. ovale* | *H. sapiens* | -- | X99790 |
| *P. simiovale* | *M. sinica* | Sri Lanka | AB287286 |
| *P. simiovale* | *M. sinica* | Sri Lanca | AB287287 |
| *P. vivax* | *H. sapiens* | India | JQ627158 |
| *P. vivax* | *H. sapiens* | -- | U03079 |
| *P. vivax* | *H. sapiens* | Thailand | U07367 |
| *P. vivax* | *H. sapiens* | -- | U83877 |
| *P. vivax* | *H. sapiens* | Thailand | U93233 |
| *P. vivax* | *H. sapiens* | -- | X13926 |
| *P. coatneyi* (B136co) | *An. balabacensis* | Sabah, Malaysia | MF582546 |
| *P. coatneyi* (PD991co) | *An. balabacensis* | Sabah, Malaysia | MF582547 |
| *P. coatneyi* (PD1015co) | *An. balabacensis* | Sabah, Malaysia | MF582548 |
| *P. cynomolgi* (A220cy) | *An. balabacensis* | Sabah, Malaysia | MF582549 |
| *P. cynomolgi* (B124cy) | *An. balabacensis* | Sabah, Malaysia | MF582550 |
| *P. cynomolgi* (PD1430cy) | *An. balabacensis* | Sabah, Malaysia | MF582551 |
| *P. cynomolgi* (PD1538cy) | *An. balabacensis* | Sabah, Malaysia | MF582552 |
| *P. fieldi* (A125fi) | *An. balabacensis* | Sabah, Malaysia | MF582553 |
| *P. fieldi* (B124fi) | *An. balabacensis* | Sabah, Malaysia | MF582554 |
| *P. fieldi* (PD1538fi) | *An. balabacensis* | Sabah, Malaysia | MF582555 |
| *P. inui* (A125i) | *An. balabacensis* | Sabah, Malaysia | MF582556 |
| *P. inui* (A220i) | *An. balabacensis* | Sabah, Malaysia | MF582557 |
| *P. inui* (PD1050i) | *An. balabacensis* | Sabah, Malaysia | MF582558 |
| *P. inui* (PD1217i) | *An. balabacensis* | Sabah, Malaysia | MF582559 |
| *P. inui* (PD1430i) | *An. balabacensis* | Sabah, Malaysia | MF582560 |
| *P. inui* (SWD3i) | *M. fascicularis* | Sabah, Malaysia | MF582561 |
| *P. knowlesi* (F36k) | *H. sapiens* | Sabah, Malaysia | MF582562 |
| *P. knowlesi* (KM12k) | *H. sapiens* | Sabah, Malaysia | MF582563 |
| *P. knowlesi* (PD615k) | *An. balabacensis* | Sabah, Malaysia | MF582564 |
| *P. knowlesi* (PD1217k) | *An. balabacensis* | Sabah, Malaysia | MF582565 |
| *P. knowlesi* (SWD5k) | *M. fascicularis* | Sabah, Malaysia | MF582566 |
| -- not specified | |  |  |
